# Development and validation of nutrient estimates based on a food-photographic record in Japan

**DOI:** 10.1186/s12937-020-00615-y

**Published:** 2020-09-18

**Authors:** Keigo Saeki, Naoto Otaki, Maiko Kitagawa, Nobuhiro Tone, Ribeka Takachi, Rika Ishizuka, Norio Kurumatani, Kenji Obayashi

**Affiliations:** 1 Department of Epidemiology, Nara Medical University School of Medicine, 840 Shijocho, Kashihara, Nara, 634-8521 Japan; 2 Department of Food Sciences and Nutrition, Mukogawa Women’s University, Hyogo, Japan; 3 Otemae College of Nutrition, Osaka, Japan; 4 Oura clinic, Nara, Japan; 5 Center for Academic Industrial and Governmental Relations, Nara Medical University School of Medicine, Nara, Japan; 6 Takatori corporation, Nara, Japan; 7 Department of Food Science and Nutrition, Nara Women’s University Graduate School of Humanities and Sciences, Nara, Japan; 8 Department of Food and Nutrition Faculty of Contemporary Human Life Science, Tezukayama University, Nara, Japan

**Keywords:** Food-photographic record, Validity, Nutrient estimate, Portion size estimation

## Abstract

**Background:**

Previous studies have reported that estimates of portion size, energy, and macronutrients such as carbohydrates, proteins, and fat based on the food-photographic record closely correlate with directly weighed values. However, the correlation based on a large sample of the test meal with the evidence of many nutrients is yet to be determined. We conducted this study to assess the correlation and difference between the food-photographic record and weighed results for 44 nutrients based on a larger number of test meals than those in previous studies.

**Methods:**

We assessed the nutrients of test meals using a food-photographic record and direct weighing and compared the results of the two methods. Twenty participants prepared a total of 1163 test meals. Each participant cooked 28–29 different kinds of dishes. Five participants cooked the same dish with their own recipes. For the most commonly consumed 41 dishes, 20 participants served a meal with their usual portion size. For the remaining 73 dishes, five participants served a meal with their usual portion size. An independent researcher weighed each ingredient and calculated the nutrients of the test meals. The participants took photographs of the test meals using a digital camera. Two independent, trained analysts measured the longitudinal and transverse diameters of the food area on the photographs of the test meals, compared the portion size with the reference photographs, and calculated the nutrients based on a database that contained reference photographs.

**Results:**

Rank correlation coefficients between estimates from the food-photographic record of each test meal and weighed results were high for portion size (*r* = 0.93), energy (*r* = 0.93), protein (*r* = 0.90), fat (*r* = 0.92), and carbohydrate (*r* = 0.94), and those for the 44 nutrients ranged from 0.78 to 0.94. We found high reproducibility between the two analysts for all the nutrients (*r* > 0.90).

**Conclusions:**

We found a high correlation and small difference between the food-photographic record method and weighed results of a large number of nutrients in many test meals.

## Introduction

A weighed dietary record maintained for multiple days is an accurate method used to estimate actual food intake, but it is impractical for an epidemiologic study because it demands a high degree of cooperation from the study participants [1]. Hence, an alternative method is needed to assess nutrient intake, which involves a lesser burden for the participants. Food photography of various portion sizes is useful for the individual to recall the amount of food consumed. The photograph-guided recall method closely correlated with the directly weighed portion size, energy intake, and nutrients [2–6].

The food-photographic record (FPR) is proposed as a more objective method than the photograph-guided recall method. In the photograph-guided recall method, the study participants recall the amount of their food consumption. However, in FPR, the study participants take food photographs, and independent analysts objectively estimate the portion size and nutrients by comparing a test meal photograph with a reference photograph of the standard portion size.

Williamson et al. conducted validation studies in a laboratory setting [7, 8]. They showed a high correlation between estimates of portion size based on FPR and independently weighed results of 453 test meals. Martin et al. assessed the correlation of estimates from FPR and weighed results in a real-life situation based on 150 test meals consumed over 3 days by 52 individuals in both dine-in and take-out settings. The estimates of energy intake based on FPR closely correlated with the energy intake calculated from the weighed values by an independent researcher in the dine-in setting (*r* = 0.93), take-out lunch setting (*r* = 0.93), and take-out dinner setting (*r* = 0.95) [9]. The estimates of energy intake based on an FPR were similar to the results of doubly labeled water (DLW) methods with a small error (− 3.7%) [10]. Several studies have provided evidence of the usefulness of FPR in school settings [11], hospitals [12], and free-living settings [13–15]. A previous study showed that the burden of the study participants to take photographs of food is relatively small [9]. The reference weighed value should be assessed by an independent researcher other than the food consumer because self-reported weighed dietary records by food consumers have led to the underestimation of energy intake by 20% [16] and protein intake by 6.4% [17] compared with objectively measured biomarkers. A few validation studies have compared the estimates from an FPR and independently assessed results [7, 9, 10].

Despite these reports, three issues remain to be clarified. First, the agreement between FPR and the weighed results was assessed using a relatively small number of test meals. Second, the correlation and difference between estimates from FPR and those of weighed results have been reported only for limited kinds of nutrients, such as energy, carbohydrates, proteins, and fat. Third, the correlation and difference between estimates from FPR and weighed results have not yet been assessed in the presence of the error derived from portion-size estimation as well as differences in recipes. Analysts in previous validation studies estimated the portion size by comparing test meal photographs and reference photographs of standard portion size. In these studies, the test meal and reference meal were cooked using the same recipe [9, 11, 12]. The agreement of estimates from FPR with weighed results, therefore, depended solely on the accuracy of the portion-size estimates. However, when the nutrients are estimated based on FPR in a real-life situation, the reference photograph of the meal cooked with the same recipe as the target meal would not be available. Therefore, the difference between estimates from FPR and the weighed value was determined not only from the portion-size estimation but also from the difference in recipes of the test meal photographs and reference photographs.

To overcome the limitation of the previous studies, we assess the correlation and difference of estimates from FPR and the weighed results for 44 kinds of nutrients using the largest number of test meals. To take into account the errors derived from portion size estimation and difference of recipe, we used the reference photographs cooked with the standard recipe, and test meals were cooked by participants with their own recipe.

## Methods

We assessed the nutrients of test meals by FPR and direct weighing and compared the results of the two methods. We investigated 44 nutrients measured in the National Health and Nutrition Survey in Japan (Supplementary list). This study complied with the Declaration of Helsinki, and the study protocol was approved by the Nara Medical University Medical Ethics Committee.

### Test meal for the validation study

**Fig. 1:**
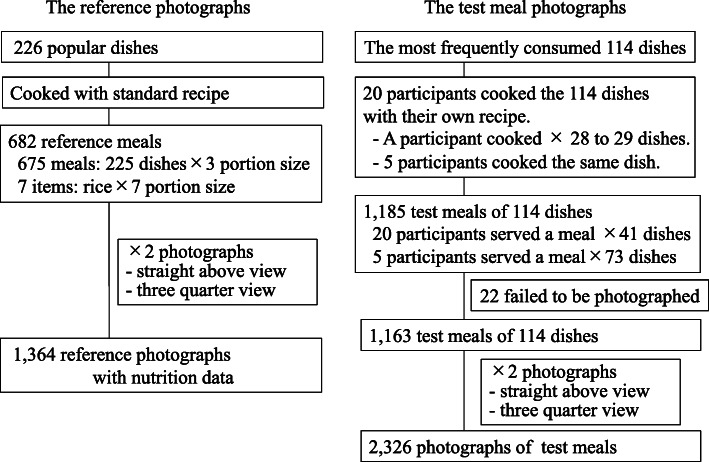
Preparation of the reference photographs and the test meal photographs

The HEIJO-KYO study comprises a sample of elderly participants from the Japanese population. In this study, 322 participants recorded their meals for three consecutive days. All participants provided written informed consent, and Nara Medical University’s Ethics Committee approved the study protocol. The mean age of participants in the HEIJO-KYO study was 71.5 years, and 47.9% were men [18]. We determined the most popular 226 dishes based on the food record. From 322 participants of the HEIJO-KYO study, we recruited 20 participants who can attend the eight cooking sessions in a laboratory setting. The mean age of the 20 participants was 67.6 years, and 90% were women. Of the 226 dishes, 114 of the most frequently consumed dishes were cooked freely along with their own recipe by 20 participants. Each participant cooked 28 to 29 different kinds of dishes. Five participants cooked the same dish with recipes of their own (Fig. 1). The number of dishes for the test meal was determined by the limitation of research resources and the research period.

Before the cooking sessions, we prepared typical foodstuffs. The participants could choose their favorite cooking ingredients and use the usual amount of these ingredients to prepare these dishes. A participant completed all the cooking procedures of a particular dish. Before and during the cooking session, the participants received no information about the standard recipe for each dish.

Accurate assessment for the amount of frequently consumed dishes, such as rice, cooked rice, and miso-soup is important to estimate habitual nutrition intake. In order to reflect the importance of frequently consumed staple food in the validation results, 20 participants served a meal with the amount of food that they would usually consume for the most commonly consumed 41 dishes. For the other 73 dishes, five participants served a meal with their usual portion size. (Fig. 1).

#### Direct weighing of nutrients

During cooking of the test meals, an independent researcher directly weighed each ingredient using a digital scale. Each meal was directly weighed, and the nutrient content was calculated based on the data derived from Standardized Tables of Food Composition in Japan, 6th edition [19].

#### Food-photographic record method

The participants who served the test meals took two photographs of each meal from a straight-above view and a three-quarter view with a 1-cm scale using a digital camera (IXY150, Canon Inc., Tokyo, Japan). After excluding 22 meals (failed to be photographed), a total of 2326 photographs of 1163 test meals were used (Fig. 1).

Two trained analysts independently estimated the portion sizes of the test meals by comparing the photographs of the test meals with the reference photographs (described below). The analysts measured the longitudinal and transverse diameters of the food area in the photographs and assessed the portion sizes using a 5-point rating scale. The portion sizes of the test meals, which were of small size, small to standard size, standard size, standard to large size, and large size corresponded to 1, 2, 3, 4, and 5, respectively. We calculated the nutrients based on the nutrition data that attached to the reference photographs. Similar to the nutrients of portion size 2 and 4, we calculated the mean value of portion size 1 and 3, and that of 3 and 5. In this analysis, we used the mean values of the nutrition estimates carried out by two independent analysts.

### Reference photographs for a food-photographic record method

**Fig. 2:**
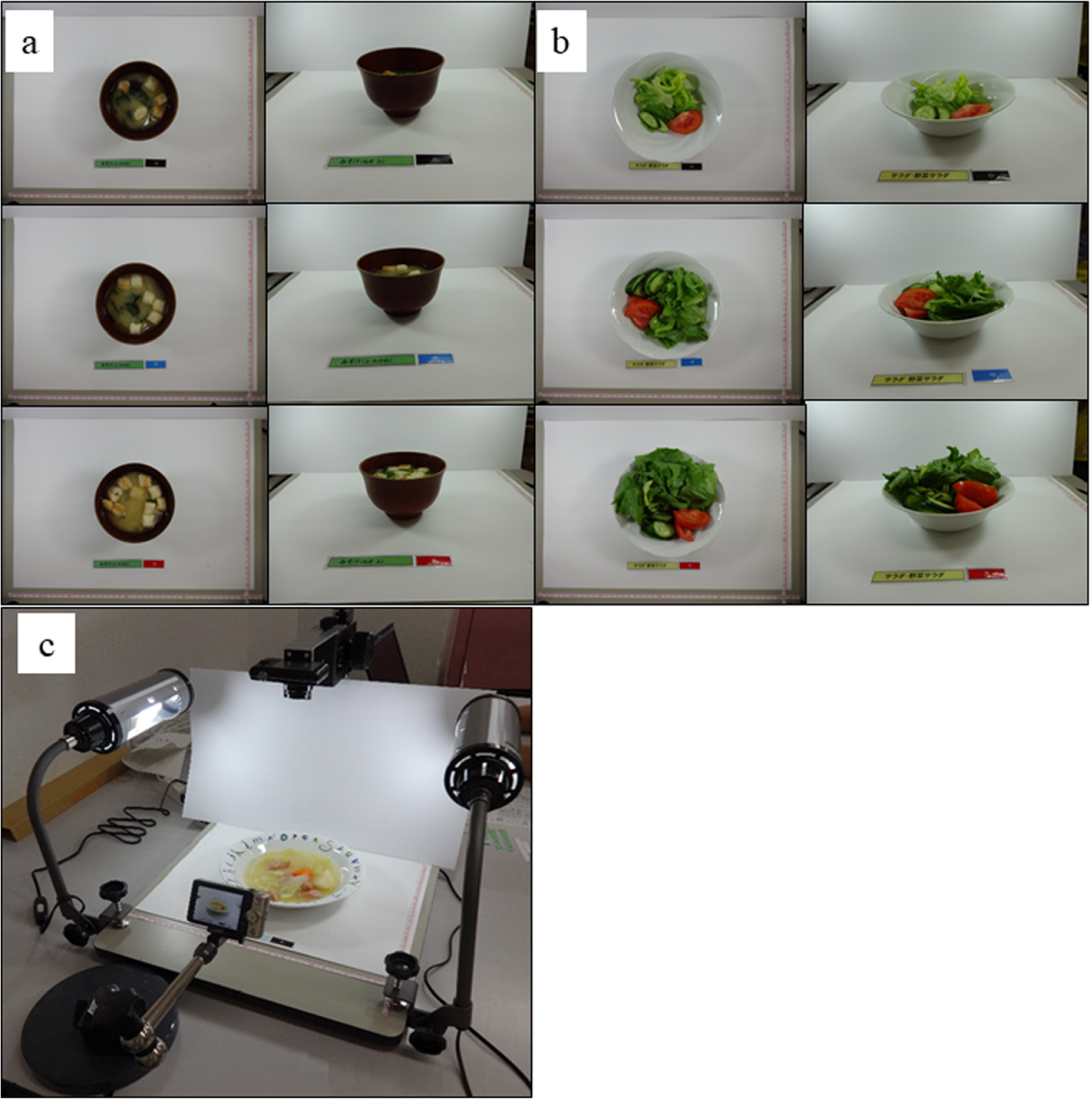
**a** Reference photographs from straight above view and three-quarter view of miso soup with three portion sizes and (**b**) those of vegetable salad with three portion sizes. **c** The apparatus used for taking photos of the reference food

We prepared reference photographs of the most popular 226 dishes that were to be used in FPR method. The reference meals with the standard portion sizes were cooked according to the typical standard recipe found in Japanese guidebooks [20–23] and served in three different portion sizes: standard size, small size (50% smaller than the standard size), and large size (50% larger than the standard size). Rice is a staple food and is more frequently consumed than other foods. To maintain estimation accuracy, we prepared seven different portion sizes equivalent to 50, 75, 100, 125, 150, 175, and 200% of the standard portion size for rice. Meanwhile, we took photographs of the 682 reference meals from a straight-above view (camera angle 90°) and a three-quarter view with a 1-cm interval scale (a total of 1364 photographs). Thus, we took 14 photographs for rice and six photographs for the other meals (Fig. 2-a and b). We used a digital scanner stand (DDS-400S, LPL Co., Ltd., Saitama, Japan) to take reference photographs (Fig. 2-c). A digital camera (IXY150, Canon Inc., Tokyo, Japan) was positioned 40 cm above the food, and another camera was set upward at a 45° angle. To decrease the influence of shadows, we illuminated both sides of each meal. After the calculation of nutrients based on the Standardized Tables of Food Composition in Japan, 6th edition, the nutrition data were attached to the reference photographs [19].

### Statistical analysis

**Fig. 3:**
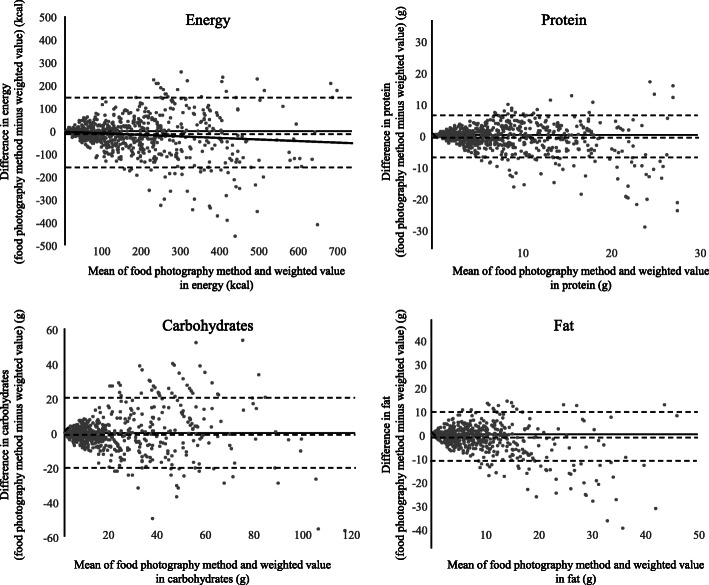
Bland-Altman plot of the food-photographic record and weighed results. Dotted lines show mean difference and 95% limits of agreement (1.96 ± SD of difference)

We presented median and interquartile ranges (IQRs) of weighed values and estimates from FPR (Tables 1, 2 and 3). The correlation between estimates from FPR and weighed values with skewed distribution was assessed using the Spearman’s rank correlation coefficient. We regarded the test items as “zero items” when both the weighed value and FPR estimate were zero. We conducted a sensitivity analysis, excluding zero items (Supplementary Table 1). We compared the proportion of difference (weighed value minus estimates from FPR) to the weighed value as a percentage difference (Table 1). We assessed the agreement between the two quantitative measurements using the Bland– Altman method. We plotted the difference between estimates from FPR minus the weighed value on the y-axis and the mean of the estimates and the weighed value on the x-axis (Fig. 3). Systematic errors of estimates based on the weighed values were assessed using the slope of the regression line (Supplementary Table 2).

**Fig. 4:**
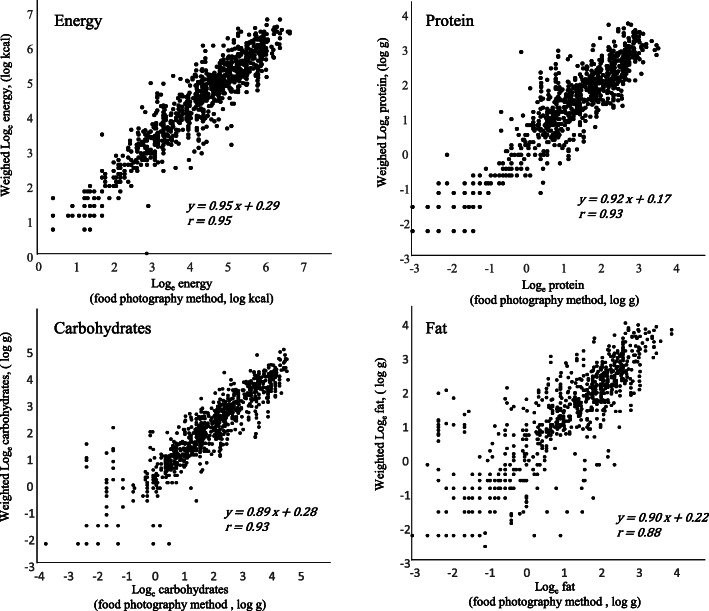
Log-linear regressions between estimates from the food-photographic record and weighed values

To correct the influence of systematic errors, prediction equations for each nutrient were developed based on the log-linear regression model (Fig. 4). Table 2 shows the parameters of log-linear regression models in 44 nutrients. The corrected value of each nutrient was calculated using the following equation: Y = e^a^ × (X)^b^, where Y is the corrected value, a is an intercept of the regression line, b is a regression coefficient, and X is the estimate based on FPR. The goodness-of-fit was presented based on the coefficient of the determinant of the regression lines (R^2^), and we compared the corrected estimates from FPR with the weighed value based on the proportion of difference and rank correlation (Table 2).

Statistical tests with two-sided *P* values < 0.05 were considered significant. All analyses were conducted using SPSS version 24.

## Results

Based on the rank correlation among 1163 test meals, as presented in Table 1, estimates from FPR closely correlated with the weighed value for the portion size (*r* = 0.93, *P* < 0.001), energy (*r* = 0.93, *P* < 0.001), macronutrients such as protein (*r* = 0.90, *P* < 0.001), fat (*r* = 0.92, *P* < 0.001), and carbohydrates (*r* = 0.94, *P* < 0.001). The correlation coefficient of all nutrients ranged from 0.78 (sodium and α-carotene) to 0.94 (carbohydrates). Estimates from FPR were lower than the weighed value for energy (relative difference median: − 10.0%; interquartile range [IQR]: − 31.3. 18.4%), protein (− 6.3%; − 29.5 to 26.5%), fat (− 7.3%; − 38.3 to 25.0%), and carbohydrates (− 8.8%; − 32.0 to 24.3%, Table 1). Exclusion of zero items substantially decreased the correlation coefficient for vitamin B_12_ and iodine, but the correlations between estimates from FPR and weighed values for these nutrients remained moderate (Supplementary Table 1).

**Table 1:** Nutrients between directly weighed value and estimates based on the food-photographic record among 1163 test meals

	weighed value median (IQR)	food photography median (IQR)	% difference^a^ median (IQR)	correlation coefficient^b^	P for correlation‡
Portion size, g	104.6 (31.2, 210.6)	83.0 (30, 188.6)	− 10.6 (−31.3, 15.3)	0.93	< 0.001
Energy, kcal	91.0 (26.0, 202.0)	79.5 (23, 194)	−10.0 (−31.8, 18.4)	0.93	< 0.001
Protein, g	4.6 (1.6, 8.5)	4 (1.5, 9.2)	−6.3 (−29.5, 26.5)	0.90	< 0.001
Fat, g	2.5 (0.3, 8)	2.1 (0.3, 6.9)	−7.3 (−38.3, 25.0)	0.92	< 0.001
Triglyceride, g	2.1 (0.1, 7)	1.7 (0.1, 6.4)	−12.5 (−42.2, 17.7)	0.90	< 0.001
SFA, g	0.5 (0.02, 1.96)	0.4 (0.03, 1.53)	−13.6 (−43.2, 20.4)	0.89	< 0.001
MUFA, g	0.6 (0.01, 2.86)	0.5 (0.02, 2.47)	−13.9 (− 41.4, 21.1)	0.91	< 0.001
PUFA, g	0.4 (0.03, 2.02)	0.5 (0.04, 2.13)	−5.8 (− 35.4, 33.3)	0.90	< 0.001
Cholesterol, mg	5.2 (0, 37.83)	3.3 (0, 32.54)	−20.1 (−45.5, 24.7)	0.87	< 0.001
Carbohydrate, g	6.8 (2.5, 20.5)	5.6 (2.1, 18.4)	−8.8 (−32.0, 24.3)	0.94	< 0.001
Total dietary fiber, g	0.8 (0.3, 2)	0.8 (0.2, 1.9)	−7.8 (− 33.8, 21.5)	0.90	< 0.001
Water soluble, g	0.2 (0.01, 0.47)	0.1 (0, 0.4)	−15.5 (− 41.6, 14.9)	0.91	< 0.001
Water insoluble, g	0.6 (0.1, 1.4)	0.5 (0.07, 1.46)	−8.0 (−33.8, 21.1)	0.91	< 0.001
Sodium, mg	322.6 (176, 534.4)	311.7 (170, 535.3)	−6.5 (−32.9, 31.5)	0.78	< 0.001
Potassium, mg	175.4 (68.9, 339)	151.5 (62.3, 300)	−9.4 (−33.3, 19.1)	0.88	< 0.001
Calcium, mg	25.7 (10.9, 51.7)	23.5 (9, 45.1)	−10.4 (−34.5, 21.5)	0.88	< 0.001
Magnesium, mg	15.4 (7.2, 29.7)	16.1 (7, 29.6)	−6.7 (−29.2, 22.5)	0.87	< 0.001
Phosphorus, mg	72.6 (28.9, 130.1)	65.8 (28, 129.5)	−6.3 (− 29.4, 25.5)	0.86	< 0.001
Iron, mg	0.5 (0.2, 1)	0.5 (0.2, 1)	−6.5 (−28.6, 27.2)	0.87	< 0.001
Zinc, mg	0.4 (0.1, 1)	0.4 (0.1, 0.9)	−9.1 (− 33.3, 22.7)	0.87	< 0.001
Copper, mg	0.06 (0.02, 0.12)	0.06 (0.01, 0.12)	−7.1 (−28.8, 26.4)	0.90	< 0.001
Manganese, mg	0.07 (0.02, 0.26)	0.08 (0.02, 0.24)	−7.0 (− 33.3, 27.4)	0.90	< 0.001
Iodine, μg	0.5 (0, 4.08)	0.6 (0, 4.04)	−9.5 (−43.0, 67.3)	0.85	< 0.001
Selenium, μg	0.9 (0, 5.4)	1.2 (0, 5.44)	−1.5 (−32.2,51.5)	0.92	< 0.001
Chromium, μg	0.2 (0, 0.68)	0.2 (0, 0.68)	−8.6 (−41.3, 43.2)	0.88	< 0.001
Molybdenum, μg	3.3 (0, 10.86)	3.3 (0, 10.13)	−3.8 (− 32.0, 49.5)	0.89	< 0.001
Retinol, μg	0 (0, 13.15)	0 (0, 7.88)	−29.1 (−68.9, 21.8)	0.80	< 0.001
α-carotene, μg	0 (0, 46.91)	0 (0, 4.45)	−8.5 (− 65.0, 42.9)	0.78	< 0.001
β-carotene, μg	35 (0, 446.25)	28.5 (0, 540)	−11.5 (−51, 27.2)	0.87	< 0.001
Cryptoxanthin, μg	0 (0, 2.19)	0 (0, 2.38)	−15.4 (−60.7, 25.0)	0.82	< 0.001
Vitamin D, μg	0 (0, 0.4)	0.03 (0, 0.38)	−20.1 (−48.5, 23.1)	0.83	< 0.001
α-tocopherol, mg	0.3 (0.08, 0.86)	0.3 (0.07, 0.88)	−6.9 (−34.3, 31.5)	0.91	< 0.001
β-tocopherol, mg	0 (0, 0.05)	0 (0, 0.06)	6.6 (−31.3, 80.0)	0.89	< 0.001
γ-tocopherol, mg	0.3 (0, 1.78)	0.2 (0, 2.25)	−1.1 (−33.7, 49.6)	0.87	< 0.001
δ-tocopherol, mg	0.03 (0, 0.4)	0.01 (0, 0.51)	0.0 (−37.7, 75.3)	0.84	< 0.001
Vitamin K, μg	8.5 (0.7, 22.2)	7.5 (0.4, 22)	−12.3 (−41.3, 22.5)	0.90	< 0.001
Vitamin B1, mg	0.05 (0.03, 0.11)	0.05 (0.02, 0.1)	−9.2 (−34.4, 25.6)	0.87	< 0.001
Vitamin B2, mg	0.06 (0.02, 0.14)	0.05 (0.02, 0.13)	−8.3 (−33.3, 20.0)	0.92	< 0.001
Niacin, mg	0.9 (0.2, 2.2)	0.8 (0.2, 2.4)	−6.5 (−33.1, 26.8)	0.89	< 0.001
Vitamin B6, mg	0.06 (0.02, 0.15)	0.06 (0.02, 0.16)	−7.7 (−33.2, 25.0)	0.87	< 0.001
Vitamin B12, μg	0.3 (0, 0.61)	0.2 (0, 0.52)	−11.9 (−42.3, 33.3)	0.86	< 0.001
Folate, μg	18.4 (6.4, 34.9)	17.2 (5.7, 33)	−7.4 (−33.8, 24.8)	0.87	< 0.001
Pantothenic acid, mg	0.3 (0.08, 0.75)	0.3 (0.08, 0.7)	−9.0 (−31.1, 23.1)	0.89	< 0.001
Biotin, μg	1.4 (0, 3.45)	1.5 (0, 3.67)	−0.1 (−31.0, 48.2)	0.87	< 0.001
Vitamin C, mg	3.1 (0, 11.12)	2.6 (0, 9.35)	−18.2 (−49.5, 24.1)	0.91	< 0.001

*SFA* Saturated fatty acid, *MUFA* Monounsaturated fatty acid, *PUFA* Polyunsaturated fatty acid

^a^(food photography method - weighed value) / (weighed value) × 100

^b^Spearman’s rank correlation coefficient

^‡^ *P* value for the correlation

Based on the Bland–Altman plot, we found a higher variance in the difference (estimates from FPR minus weighed value) along with the increase in the mean value of energy, protein, carbohydrates, and fat (Fig. 3). The regression line in the plot exhibited a significantly negative slope in the case of energy (regression coefficient: − 0.072, 95% confidence interval: − 0.103 to − 0.042), proteins (− 0.132, − 0.168 to − 0.095), and fat (− 0.309, − 0.348 to − 0.269), except for carbohydrates wherein we found a positive slope (0.030, − 0.001 to 0.061). More underestimation was significantly associated with a higher mean for most nutrients (Supplementary Table 2).

**Table 2:** Prediction equations to correct estimates from the food-photographic record

	No. of meals	Regression coefficient (b)^a^	Intercept (a)^a^	Correlation coefficient^b^ (r_p_)	R^2c^	Corrected estimates median (IQR)	% difference^d^ after correction median (IQR)
Portion size, g	1163	0.99	0.17	0.95	0.90	93.6 (34.2, 210.7)	0.5 (−22.7, 31.1)
Energy, kcal	1163	0.95	0.29	0.95	0.90	87.0 (26.7, 203.5)	−0.4 (−24.3, 29.6)
Protein, g	1163	0.92	0.17	0.93	0.87	4.2 (1.7, 9.1)	1.7 (−25.0, 35.8)
Fat, g	992	0.90	0.22	0.88	0.77	3.2 (0.9, 8.3)	1.7 (−29.4, 50.9)
Triglyceride, g	871	0.86	0.32	0.87	0.76	4.1 (1.2, 8.4)	4.1 (−28.3, 46.5)
SFA, g	966	0.94	0.20	0.91	0.83	0.8 (0.1, 2.2)	9.5 (−29.2, 56.9)
MUFA, g	928	0.91	0.16	0.91	0.82	1.1 (0.2, 3.2)	4.0 (−31.0, 53.2)
PUFA, g	962	0.85	0.02	0.90	0.80	0.9 (0.2, 2.2)	5.6 (−28.5, 55.2)
Cholesterol, mg	658	0.66	1.28	0.74	0.55	29.9 (13.9, 58.3)	2.5 (−39.0, 71.4)
Carbohydrate, g	1147	0.89	0.28	0.93	0.86	6.2 (2.6, 17.8)	−0.5 (−24.9, 34.8)
Total dietary fiber, g	931	0.80	0.10	0.83	0.69	1.2 (0.6, 2.0)	−0.1 (−23.8, 31.8)
Water soluble, g	849	0.77	−0.18	0.83	0.68	0.3 (0.1, 0.5)	−1.8 (−26.1, 31.9)
Water insoluble, g	878	0.79	0.02	0.83	0.69	0.9 (0.4, 1.5)	−3.1 (−24.1, 30.6)
Sodium, mg	1163	0.92	0.45	0.85	0.72	302.3 (173.4, 496.3)	−7.1 (−30.9, 29.0)
Potassium, mg	1163	0.89	0.65	0.91	0.82	167.9 (76.1, 308.4)	2.7 (−24.0, 33.5)
Calcium, mg	1163	0.89	0.43	0.91	0.82	25.9 (11.0, 46.4)	0.6 (−24.9, 37.7)
Magnesium, mg	1154	0.87	0.39	0.88	0.77	16.7 (8.1, 28.4)	−0.9 (−22.5, 29.6)
Phosphorus, mg	1163	0.91	0.41	0.91	0.83	68.5 (31.4, 127.1)	0.3 (−24.9, 32.7)
Iron, mg	1128	0.88	−0.07	0.89	0.79	0.6 (0.2, 0.9)	−3.5 (−25.2, 32.0)
Zinc, mg	1154	0.91	−0.02	0.90	0.81	0.4 (0.2, 0.9)	−0.2 (−26.6, 32.6)
Copper, mg	1153	0.88	− 0.34	0.91	0.82	0.06 (0.02, 0.11)	−3.2 (−23.5, 28.7)
Manganese, mg	1033	0.86	−0.30	0.88	0.78	0.1 (0.03, 0.23)	−1.8 (−26.6, 33.8)
Iodine, μg	777	0.46	0.27	0.50	0.25	1.9 (1.0, 3.6)	−3.4 (−69.1, 137.8)
Selenium, μg	805	0.83	0.11	0.77	0.59	2.2 (1.1, 7.1)	−8.6 (−39.8, 55.2)
Chromium, μg	699	0.67	−0.16	0.68	0.46	0.5 (0.3, 0.9)	−5.5 (−40.4, 52.1)
Molybdenum, μg	822	0.74	0.48	0.76	0.58	6.4 (3.5, 13.5)	−6.1 (−36.2, 48.5)
Retinol, μg	433	0.71	0.97	0.74	0.55	19.2 (5.1, 35.6)	−5.2 (−38.0, 74.3)
α-carotene, μg	412	0.86	0.16	0.85	0.73	107.0 (2.1, 194.5)	−19.3 (−49.4, 28.6)
β-carotene, μg	784	0.88	0.68	0.88	0.77	132.5 (29.1, 681.6)	3.7 (−37.9, 67.8)
Cryptoxanthin, μg	381	0.89	0.19	0.84	0.70	5.4 (2.0, 12.7)	−4.8 (− 37.1, 35.6)
Vitamin D, μg	512	0.93	−0.01	0.83	0.69	0.4 (0.2, 0.9)	−11.3 (−36.3, 39.4)
α-tocopherol, mg	1043	0.90	−0.07	0.89	0.79	0.4 (0.1, 0.9)	2.0 (−27.9, 40.3)
β-tocopherol, mg	544	0.84	−0.62	0.69	0.48	0.05 (0.03, 0.08)	−5.6 (−40.5, 56.6)
γ-tocopherol, mg	806	0.80	−0.08	0.85	0.72	1.1 (0.2, 2.2)	7.0 (−32.2, 55.2)
δ-tocopherol, mg	565	0.83	−0.33	0.79	0.63	0.4 (0.2, 0.6)	3.7 (−37.3, 48.6)
Vitamin K, μg	900	0.85	0.43	0.88	0.77	13.7 (4.8, 28)	−3.1 (−31.4, 36.6)
Vitamin B_1_, mg	1142	0.89	−0.24	0.89	0.79	0.05 (0.03, 0.10)	1.8 (−25.6, 42.5)
Vitamin B_2_, mg	1162	0.95	−0.03	0.93	0.87	0.06 (0.02, 0.14)	3.1 (−22.6, 37.1)
Niacin, mg	1162	0.90	0.04	0.92	0.84	0.8 (0.3, 2.3)	3.4 (−25.0, 39.1)
Vitamin B_6_, mg	1153	0.90	−0.22	0.89	0.80	0.06 (0.02, 0.15)	−0.4 (−27.1, 35.0)
Vitamin B_12_, μg	804	0.61	−0.37	0.56	0.31	0.4 (0.3, 0.6)	−15.9 (−42.3, 29.3)
Folate, μg	1135	0.85	0.51	0.89	0.79	18.5 (7.8, 32.6)	2.1 (−24.6, 40.6)
Pantothenic acid, mg	1140	0.90	−0.06	0.90	0.81	0.3 (0.1, 0.7)	1.0 (−24.6, 32.4)
Biotin, μg	831	0.84	0.14	0.78	0.60	2.3 (1.5, 3.9)	−0.2 (−32.2, 54.8)
Vitamin C, mg	800	0.81	0.46	0.83	0.69	6.8 (3.3, 14.1)	−4.0 (− 33.3, 51.9)

*SFA* Saturated fatty acid, *MUFA* Monounsaturated fatty acid, *PUFA* Polyunsaturated fatty acid

^a^Prediction Equation: Y = e^a^ × (X)^b^, Y: directly weighed value, X: estimates based on a food-photographic record

^b^Pearson’s correlation coefficient between log weighed value and estimates from a food-photographic record

^c^The values of R^2^ indicate the percentage of the variance of log-Y accounted by equations

^d^(Corrected value from a food-photographic record - weighed value)/(weighed value) × 100

Log-transformed estimates from FPR and log-transformed weighed values exhibited a strong correlation for energy (*r* = 0.95), protein (*r* = 0.93), carbohydrates (*r* = 0.93), and fat (*r* = 0.88) (Fig. 4).

Based on the coefficient of determination (R^2^), the variance in log-transformed weighed values can be explained by equations over 85% in portion size (90%), energy (90%), protein (87%), vitamin B_2_ (87%), and carbohydrates (86%) (Table 2). In contrast, the coefficient of determination was low for iodine (25%) and vitamin B_12_ (31%). Corrections using these prediction equations decreased the difference between weighed value and estimates in energy (median of relative difference: − 0.4%), protein (1.7%), carbohydrates (− 0.5%), and fat (1.7%) (Table 2).

We found that there was high reproducibility and the small difference between the estimates of the two independent analysts. The median value of their estimates was similar, and the rank correlation coefficients between the estimates of the two analysts were higher than 0.9 for all the 44 nutrients (Table 3).

**Table 3:** Reproducibility of estimates based on a food-photographic record between two independent analysts

	Analyst 1	Analyst 2	Correlation
	median (IQR)	median (IQR)	coefficient^a^
Portion size, g	81.5 (30.0, 184.5)	83.0 (30.0196.6)	0.95
Energy, kcal	78.0 (22.5, 184)	79.5 (24.0, 199.5)	0.96
Protein, g	3.9 (1.5, 8.6)	4.1 (1.5, 9.3)	0.95
Fat, g	2.8 (0.6, 7.7)	2.8 (0.6, 8.3)	0.97
Triglyceride, g	2.8 (0.6, 7.5)	3.1 (0.6, 8.2)	0.97
SFA, g	0.5 (0.1, 1.7)	0.6 (0.1, 1.9)	0.98
MUFA, g	0.9 (0.1, 2.9)	0.9 (0.1, 3.3)	0.97
PUFA, g	0.8 (0.1, 2.3)	0.8 (0.1, 2.5)	0.97
Cholesterol, mg	22.0 (6.0, 53.1)	24.0 (6.0, 64.9)	0.96
Carbohydrate, g	5.6 (2.1, 18.2)	5.7 (2.1, 18.4)	0.97
Total dietary fiber, g	1.1 (0.5, 2.0)	1.1 (0.5, 2.2)	0.96
Water soluble, g	0.2 (0.1, 0.5)	0.3 (0.1, 0.5)	0.97
Water insoluble, g	0.8 (0.3, 1.6)	0.8 (0.4, 1.6)	0.97
Sodium, mg	303.0 (160.0, 494.0)	311.7 (170.0, 546.7)	0.92
Potassium, mg	150.5 (62.3, 298.2)	152.1 (65.3, 302.3)	0.95
Calcium, mg	23.4 (8.9, 44.0)	24.0 (9.0, 46.8)	0.94
Magnesium, mg	15.3 (6.8, 27.0)	16.1 (7.0, 30.8)	0.93
Phosphorus, mg	65.8 (26.0, 123.0)	67.1 (28.0, 138.0)	0.94
Iron, mg	0.5 (0.2, 0.9)	0.6 (0.2, 1.0)	0.93
Zinc, mg	0.4 (0.1, 0.9)	0.4 (0.1, 1.0)	0.95
Copper, mg	0.05 (0.01, 0.12)	0.06 (0.01, 0.12)	0.95
Manganese, mg	0.09 (0.02, 0.26)	0.10 (0.02, 0.28)	0.97
Iodine, μg	2.1 (0.6, 8.0)	2.1 (0.6, 8.7)	0.98
Selenium, μg	2.2 (0.9, 8.1)	2.2 (0.9, 9.9)	0.97
Chromium, μg	0.5 (0.2, 0.9)	0.6 (0.2, 1.2)	0.97
Molybdenum, μg	6.4 (2.5, 17.5)	6.5 (2.9, 17.5)	0.97
Retinol, μg	13.5 (2.5, 35.0)	16.8 (2.5, 42.5)	0.97
α-carotene, μg	112.0 (1.1, 280.0)	125.0 (1.1, 375.2)	0.99
β-carotene, μg	95.8 (14.2, 770.0)	111.3 (12.6, 771.6)	0.97
Cryptoxanthin, μg	4.5 (1.0, 11.7)	4.9 (1.5, 13.6)	0.96
Vitamin D, μg	0.3 (0.2, 0.8)	0.4 (0.2, 0.9)	0.94
α-tocopherol, mg	0.3 (0.1, 0.9)	0.3 (0.1, 0.9)	0.94
β-tocopherol, mg	0.06 (0.03, 0.09)	0.06 (0.03, 0.10)	0.97
γ-tocopherol, mg	0.9 (0.1, 2.7)	0.9 (0.2, 2.9)	0.97
δ-tocopherol, mg	0.4 (0.2, 0.8)	0.5 (0.2, 0.8)	0.98
Vitamin K, μg	12.0 (3.8, 25.2)	12.7 (3.8, 29.9)	0.96
Vitamin B_1_, mg	0.05 (0.02, 0.10)	0.05 (0.02, 0.10)	0.95
Vitamin B_2_, mg	0.05 (0.02, 0.13)	0.05 (0.02, 0.14)	0.94
Niacin, mg	0.8 (0.2, 2.1)	0.8 (0.2, 2.4)	0.94
Vitamin B_6_, mg	0.06 (0.02, 0.14)	0.06 (0.02, 0.15)	0.95
Vitamin B_12_, μg	0.3 (0.2, 0.7)	0.4 (0.2, 0.7)	0.94
Folate, μg	17.0 (5.9, 32.0)	18.0 (6.3, 34.8)	0.96
Pantothenic acid, mg	0.28 (0.08, 0.60)	0.29 (0.08, 0.73)	0.96
Biotin, μg	2.3 (1.2, 4.3)	2.5 (1.3, 4.6)	0.96
Vitamin C, mg	5.4 (2.1, 12.5)	6.0 (2.2, 14.0)	0.98

*SFA* Saturated fatty acid, *MUFA* Monounsaturated fatty acid, *PUFA* Polyunsaturated fatty acid

^a^Spearman’s rank correlation coefficient

## Discussion

We assessed the agreement between the estimates from FPR and independently weighed results based on 1163 freely cooked test meals. We found a relatively high correlation in 44 kinds of nutrients (Spearman’s rank correlation 0.78, 0.94). In some nutrients, we found a difference of over 20%; however, corrected nutrient estimates using the log-linear regression model showed a difference within 10%. To the best of our knowledge, this is the first study to report the correlation and difference between estimates from FPR and weighed results of major nutrients from a larger number of test meals than those in previous studies.

This study expanded the generalizability of previous validation studies of estimates from FPR. Previous studies have reported a high correlation between estimates from FPR and weighed results for portion size, energy, and macronutrients, including fat, protein, and carbohydrates. However, we need further evidence based on a larger number of test meals on many nutrition. Based on the estimates of 1163 test meals from 114 dishes, we found a high correlation of estimates from FPR and directly weighed results for 44 nutrients.

Another advantage of this study lies in using test meals cooked with the participant’s own recipe and reference photographs of food cooked using standard recipes. Previous studies compared photographs of test and reference meals cooked with the same recipe. In such a situation, the error of nutrition estimation arises only from the portion-size estimation. When we apply FPR to real-life situations, a reference image of the same meal cooked with the same recipe is not available. The error derived from the difference in the recipe that remains unknown. To the best of our knowledge, only one study using a small number of test meals assessed the correlation between estimates of FPR and weighed results in energy and macronutrients in the presence of two kinds of errors, but the weighed value was not independently assessed by an individual other than the food consumer [13]. In this study, two analysts were also involved, and they estimated the nutrient content of 1163 test meals cooked using a participant’s recipe using FPR and photographs of reference meals cooked using the standard recipe. The analysts did not take any decision regarding the difference in the recipe. Therefore, the difference between the standard recipe and participant’s own recipe would lessen the correlation between nutrition estimates of FPR and weighed results. The novelty of this study is that the nutritional estimates from FPR showed a high correlation with the weighed result, even when the effects of both the error due to the difference in the recipe and the error in estimating the portion size were considered.

We propose estimation using equations to correct the influence of systematic errors in FPR. Some previous studies have reported that estimates provided based on FPR have led to an underestimation of the weighed results. A previous validation study conducted by Martin et al. showed that estimates from FPR resulted in an underestimation of the weighed energy intake by 4.7% (*P* = 0.046) in laboratory settings and 5.5% (*P* = 0.076) in free-living settings [9]. Estimates of energy intake based on the FPR were 6.4% lower than the results of the DLW (2360 vs. 2208 kcal/day, *P* = 0.16) [10]. Other validation studies also reported lower estimates from FPR in free-living [13] and school settings [14]. In this study, we found a higher variance in the difference (estimates from FPR minus weighed value) along with the increase in the mean value in the Bland–Altman plot. This is reasonable because of the characteristics of the random error. However, a significant slope of the plot suggested the existence of systematic error due to over- or underestimation. Therefore, we proposed the use of estimation equations based on a log-linear regression model with a high coefficient of determination for the portion size (R^2^ = 0.90), energy (R^2^ = 0.90), protein (R^2^ = 0.87), carbohydrates (R^2^ = 0.86), and fat (R^2^ = 0.77, Table 2). By correcting the values using estimation equations, systematic errors were reduced according to the change in the median percentage difference before and after correction in the case of portion size (− 10.6 to 0.5%), energy (− 10.0 to − 0.4%), protein (− 6.3 to 1.7%), carbohydrates (− 8.8 to − 0.5%), and fat (− 7.3 to 1.7%). We recommend using these equations to correct FPR-based estimates in clinical practice and epidemiologic studies among the elderly people in Japan. Further studies are needed so that this data could be generalized to include young participants and for other countries.

Difficulties in estimating the kind and quantity of food used for soup stock may partly explain the reduction of correlation between estimates from FPR and weighed results. We found a reduced correlation for some nutrients after the exclusion of zero items (Supplementary Table 1). The number of zero items does not fully explain the reason for the reduction in the correlation coefficient because the correlation coefficients in the case of some nutrients such as cryptoxanthin and α-carotene remained unchanged despite the large number of zero items. The largest reduction in correlation was found for iodine (0.21; before exclusion: 0.85, after exclusion: 0.64) and in vitamin B_12_ (0.20; before exclusion: 0.85, after exclusion: 0.66). Iodine intake among Japanese individuals is higher than that of people in other counties because of the higher consumption of seaweeds such as Kombu [24]. Dried bonito shavings and dried small sardines also contain vitamin B_12_ [25]. They are used for soup stock in Japan and removed before serving. Therefore, we may underestimate these foods by FPR because they are not found in the served plate. Foods used for making Japanese soup stocks may explain the decreased correlation between estimates from FPR and weighed results for iodine and vitamin B_12_.

In addition, FPR method offers two major advantages over a weighed dietary record. First, the use of a digital device provides objective information about the timing of the meal, which is essential to the investigation of the association between circadian biological rhythms and nutrition intake. Gill et al. assessed eating-fasting rhythms using food photographs installed in a smartphone [26]. Some previous studies suggested that misalignment of circadian rhythm may decrease insulin sensitivity and leptin suppression [27], and time-restricted feeding is associated with a lower incidence of obesity compared with ad libitum diet with the same caloric intake in mice [28]. Another advantage is the decreased burden to the study participants when compared to the weighed dietary record. Martin et al. reported that 85% of the participants found it comfortable to take photographs of foods and to send data using a cellphone [9]. Results from a repeated assessment of nutrition intake based on FPR may be an alternative to a food frequency questionnaire.

Although we found a high correlation and small difference between estimates from FPR and weighed results, we should pay attention to the overestimation of the agreement as a measure to estimate the nutrition intake of the study participants in real-life settings for three reasons. First, this study compared the estimated nutrients by test meals. To estimate the daily nutrition intake of study participants, we should sum up the estimates of the meals consumed in the day and then subtract an estimate of plate waste. Second, the test meal photographs were recorded by participants under the supervision of the research staff. The quality of the record may deteriorate in real-life settings. Third, we prepared a variety of foods for the cooking sessions, and the participants could choose the type of food and amount of cooking ingredients. However, the variability of the cooking recipe may be reduced compared with real-life settings because of the lack of foodstuffs and the influence of the research staff who supervised the cooking procedure.

This study had several limitations. First, the food portion size in the photographs could be estimated only when the database contained that particular meal. To apply FPR in the epidemiologic study, we should expand the reference photograph database to cover most meals that the participants commonly consume. Second, the test meals in this study do not include soft drinks, alcoholic drinks, and sweet products. However, the photographic record of the food product with a nutritional component label will be useful for estimating its nutrient content. Third, we did not assess some kinds of food, such as fruits and vegetables, entrees, and desserts. Fourth, the agreement between FPR and weighed results will depend on the characteristics of the cook, such as age, gender, and dietary culture. We need to undertake further studies if we want to generalize our results to include younger generations and for different cultures.

## Conclusions

We assessed the agreement between the estimates from FPR and independently weighed values of portion size based on many freely cooked test meals in laboratory settings and found a relatively strong correlation (Spearman’s rank correlation coefficient > 0.75) in all the 44 nutrients considered in the study.

## Supplementary information

**Additional file 1: Table S1.** Nutrients between directly weighed value and estimates based on a food-photographic record after exclusion of zero items.

**Additional file 2: Table S2.** Agreement between weighed value and estimates from a food-photographic record using Bland-Altman analysis.

**Additional file 3:** List of the 44 nutrients measured the study.

## Data Availability

The datasets used and/or analyzed during the current study are available from the corresponding author on reasonable request.

## Abbreviations

FFQ: Food frequency questionnaire
FPR: The food-photographic record
DLW: Doubly labeled water
IQR: Interquartile ranges
