# Supplementary material for: Development and validation of nutrient estimates based on a food-photographic record in Japan
Source: Nutr J. 2020 Sep 18;19:104. doi: 10.1186/s12937-020-00615-y (PMC7501716; doi:10.1186/s12937-020-00615-y)
Supplement: Supplementary file 1 — Additional file 1: Table S1. Nutrients between directly weighed value and estimates based on a food-photographic record after exclusion of zero items. [file 12937_2020_615_MOESM1_ESM.docx]

| Supplementary Table1 Nutrients between directly weighed value and estimates based on a food-photographic record after exclusion of zero items. | | | | | |
| --- | --- | --- | --- | --- | --- |
|  | no. of | weighed value | food photography |  | % difference* |
|  | meals ‡ | median (IQR) | median (IQR) | r_s_† | median (IQR) |
| Portion size, g | 1163 | 104.6 (31.2, 210.6) | 83.0 (30, 188.6) | 0.93 | -10.6 (-31.3, 16.3) |
| Energy, kcal | 1163 | 91.0 (26.0, 202.0) | 79.5 (23, 194) | 0.93 | -10.0 (-31.8, 18.9) |
| Protein, g | 1163 | 4.6 (1.6, 8.5) | 4 (1.5, 9.2) | 0.90 | -6.3 (-29.3, 26.9) |
| Fat, g | 992 | 3.8 (0.7, 9.1) | 2.8 (0.7, 8.3) | 0.88 | -6.6 (-37.5, 25.0) |
| Triglyceride, g | 871 | 4.1 (1.2, 9.1) | 3.5 (0.9, 8.2) | 0.88 | -11.0 (-39.7, 19.4) |
| SFA, g | 966 | 0.9 (0.1, 2.4) | 0.6 (0.1, 1.9) | 0.89 | -13.6 (-42.9, 21.3) |
| MUFA, g | 928 | 1.1 (0.2, 3.6) | 0.9 (0.1, 3) | 0.91 | -13.3 (-40.9, 23.1) |
| PUFA, g | 962 | 0.9 (0.2, 2.3) | 0.9 (0.1, 2.4) | 0.87 | -5.6 (-35.1, 35.0) |
| Cholesterol, mg | 658 | 27 (10.4, 76.5) | 24.1 (7.6, 65.9) | 0.83 | -16.6 (-41.2, 28.3) |
| Carbohydrate, g | 1147 | 7 (2.7, 20.9) | 5.7 (2.1, 18.4) | 0.94 | -8.2 (-31.4, 25.0) |
| Total dietary fiber, g | 931 | 1.2 (0.5, 2.4) | 1.1 (0.5, 2.1) | 0.86 | -7.4 (-33.1, 22.7) |
| Water soluble, g | 849 | 0.3 (0.1, 0.6) | 0.3 (0.1, 0.5) | 0.87 | -12.9 (-37.5, 17.2) |
| Water insoluble, g | 878 | 0.9 (0.4, 1.8) | 0.9 (0.3, 1.6) | 0.87 | -6.5 (-31.2, 24.2) |
| Sodium, mg | 1163 | 322.6 (176, 534.4) | 311.7 (170, 535.3) | 0.78 | -6.5 (-32.8, 32.7) |
| Potassium, mg | 1163 | 175.4 (68.9, 339) | 151.5 (62.3, 300) | 0.88 | -9.4 (-33.1, 19.7) |
| Calcium, mg | 1163 | 25.7 (10.9, 51.7) | 23.5 (9, 45.1) | 0.88 | -10.4 (-34.2, 21.6) |
| Magnesium, mg | 1154 | 15.6 (7.2, 29.8) | 16.1 (7, 29.7) | 0.87 | -6.7 (-29.2, 22.9) |
| Phosphorus, mg | 1163 | 72.6 (28.9, 130.1) | 65.8 (28, 129.5) | 0.86 | -6.3 (-29.3, 26.2) |
| Iron, mg | 1128 | 0.5 (0.2, 1) | 0.6 (0.2, 1) | 0.86 | -6.5 (-28.5, 27.4) |
| Zinc, mg | 1154 | 0.5 (0.1, 1) | 0.4 (0.1, 0.9) | 0.88 | -9.1 (-33.3, 23.0) |
| Copper, mg | 1153 | 0.06 (0.017, 0.121) | 0.056 (0.013, 0.12) | 0.91 | -7.1 (-28.6, 27.0) |
| Manganese, mg | 1033 | 0.102 (0.029, 0.291) | 0.097 (0.025, 0.261) | 0.88 | -7.0 (-33.3, 27.6) |
| Iodine, µg | 777 | 1.7 (0.4, 9.3) | 2.3 (0.6, 9.4) | 0.64 | -9.4 (-42.3, 68.4) |
| Selenium, µg | 805 | 2.5 (0.8, 9.8) | 2.3 (1, 9.2) | 0.80 | -1.4 (-32.1, 51.5) |
| Chromium, µg | 699 | 0.5 (0.2, 1.2) | 0.5 (0.2, 1.1) | 0.71 | -7.6 (-40.8, 43.9) |
| Molybdenum, µg | 822 | 6.5 (3, 16.4) | 6.5 (2.9, 17.5) | 0.76 | -3.8 (-32.0, 49.9) |
| Retinol, µg | 433 | 18.2 (4.7, 50.2) | 16.1 (2.5, 38) | 0.73 | -19.9 (-50.7, 32.8) |
| α-carotene, µg | 412 | 84 (1.8, 303.8) | 187.5 (2, 375.2) | 0.82 | 10.3 (-28.5, 70.3) |
| β-carotene, µg | 784 | 210.4 (25.5, 777.6) | 119.7 (21.3, 771.6) | 0.83 | -9.1 (-41.3, 33.0) |
| Cryptoxanthin, µg | 381 | 6.4 (1.6, 14.9) | 5.3 (1.7, 13.8) | 0.84 | -3.8 (-34.5, 33.3) |
| Vitamin D, µg | 512 | 0.4 (0.2, 1.1) | 0.4 (0.2, 1) | 0.80 | -13.4 (-40.0, 30.4) |
| α-tocopherol, mg | 1043 | 0.3 (0.1, 0.9) | 0.4 (0.1, 0.9) | 0.89 | -6.3 (-32.9, 32.1) |
| β-tocopherol, mg | 544 | 0.058 (0.024, 0.103) | 0.063 (0.038, 0.098) | 0.71 | 12.9 (-25.9, 84.6) |
| γ-tocopherol, mg | 806 | 0.9 (0.2, 2.5) | 1.3 (0.2, 2.9) | 0.81 | 0 (-29.0, 54.2) |
| δ-tocopherol, mg | 565 | 0.4 (0.1, 0.9) | 0.5 (0.2, 0.8) | 0.73 | 11.8 (-24.7, 86.5) |
| Vitamin K, µg | 900 | 12.9 (5.2, 28.6) | 12.9 (3.8, 29.9) | 0.84 | -10.4 (-37.5, 25.0) |
| Vitamin B_1_, mg | 1142 | 0.055 (0.026, 0.114) | 0.048 (0.021, 0.095) | 0.87 | -9.1 (-34.4, 25.7) |
| Vitamin B_2_, mg | 1162 | 0.056 (0.021, 0.139) | 0.052 (0.02, 0.129) | 0.92 | -8.3 (-33.0, 20.1) |
| Niacin, mg | 1162 | 0.9 (0.2, 2.2) | 0.8 (0.2, 2.4) | 0.89 | -6.5 (-32.8, 27.0) |
| Vitamin B_6_, mg | 1153 | 0.06 (0.022, 0.155) | 0.056 (0.02, 0.158) | 0.89 | -7.7 (-32.8, 25.0) |
| Vitamin B_12_, µg | 804 | 0.5 (0.2, 0.8) | 0.4 (0.2, 0.8) | 0.66 | -11.0 (-40.3, 34.3) |
| Folate, µg | 1135 | 19.1 (7.1, 35.7) | 17.2 (6.2, 33.5) | 0.88 | -7.4 (-33.7, 25.0) |
| Pantothenic acid, mg | 1140 | 0.3 (0.1, 0.8) | 0.3 (0.1, 0.7) | 0.90 | -9.0 (-30.7, 24.6) |
| Biotin, µg | 831 | 2.2 (1.2, 4.8) | 2.3 (1.4, 4.3) | 0.77 | -0.1 (-31.0, 48.2) |
| Vitamin C, mg | 800 | 6.9 (2.6, 17.7) | 6 (2.5, 14.9) | 0.85 | -13.1 (-41.1, 27.8) |
| SFA, Saturated fatty acid, MUFA, Monounsaturated fatty acid, PUFA, Polyunsaturated fatty acid | | | | | |
| ^*^ (Food photography method - weighed value) / (weighed value) × 100 | | | | | |
| ^†^ Spearman's rank correlation coefficient | | | | | |
| ^‡^ Zero items: items of which both weighed value and estimates based on food photography method are zero. | | | | | |
