# Supplementary material for: Development and validation of nutrient estimates based on a food-photographic record in Japan
Source: Nutr J. 2020 Sep 18;19:104. doi: 10.1186/s12937-020-00615-y (PMC7501716; doi:10.1186/s12937-020-00615-y)
Supplement: Supplementary file 2 — Additional file 2: Table S2. Agreement between weighed value and estimates from a food-photographic record using Bland-Altman analysis. [file 12937_2020_615_MOESM2_ESM.docx]

| Supplementary Table 2 Agreement between weighed value and estimates from a food-photographic record using Bland-Altman analysis. | | | | |
| --- | --- | --- | --- | --- |
|  | no. of | difference* | agreement | regression coefficient (95%CI) |
|  | meals | mean (95%CI) | limit‡ | of Bland-Altman plot⁋ |
| Portion size, g | 1163 | -19.4 (-23.5, -15.3) | (-158.0, 51.9) | -0.125 (-0.155, -0.095) |
| Energy, kcal | 1163 | -11.8 (-16.1, -7.5) | (-158.4, 63.6) | -0.072 (-0.103, -0.042) |
| Protein, g | 1163 | -0.5 (-0.7, -0.3) | (-8.0, 5.3) | -0.132 (-0.168, -0.095) |
| Fat, g | 992 | -1.2 (-1.6, -0.9) | (-11.6, 5.9) | -0.309 (-0.348, -0.269) |
| Triglyceride, g | 871 | -1.2 (-1.6, -0.9) | (-11.2, 5.7) | -0.296 (-0.339, -0.253) |
| SFA, g | 966 | -0.4 (-0.5, -0.3) | (-3.5, 3.2) | -0.171 (-0.214, -0.128) |
| MUFA, g | 928 | -0.5 (-0.7, -0.4) | (-5.0, 3.7) | -0.322 (-0.363, -0.282) |
| PUFA, g | 962 | -0.2 (-0.3, -0.1) | (-3.3, 3.4) | -0.405 (-0.454, -0.357) |
| Cholesterol, mg | 658 | -12.1 (-15.8, -8.5) | (-105.2, 36.4) | -0.337 (-0.383, -0.291) |
| Carbohydrate, g | 1147 | -0.1 (-0.7, 0.5) | (-20.3, 12.0) | 0.030 (-0.001, 0.061) |
| Total dietary fiber, g | 931 | -0.10 (-0.21, -0.07) | (-2.2, 2.9) | -0.157 (-0.206, -0.108) |
| Water soluble, g | 849 | -0.05 (-0.06, -0.03) | (-0.6, 2.2) | -0.094 (-0.142, -0.047) |
| Water insoluble, g | 878 | -0.07 (-0.12, -0.01) | (-1.7, 2.7) | -0.148 (-0.198, -0.098) |
| Sodium, mg | 1163 | -35.2 (-53.8, -16.6) | (-669.3, 283.8) | -0.252 (-0.3, -0.204) |
| Potassium, mg | 1163 | -24.2 (-32.3, -16.1) | (-299.9, 115.7) | -0.109 (-0.149, -0.068) |
| Calcium, mg | 1163 | -4.2 (-6.6, -1.8) | (-86.2, 38.9) | -0.131 (-0.181, -0.081) |
| Magnesium, mg | 1154 | -1.4 (-2.1, -0.7) | (-25.2, 12.6) | -0.093 (-0.132, -0.054) |
| Phosphorus, mg | 1163 | -5.4 (-8.3, -2.5) | (-104.8, 46.3) | -0.070 (-0.109, -0.031) |
| Iron, mg | 1128 | -0.030 (-0.055, 0.001) | (-1.0, 2.4) | -0.098 (-0.139, -0.056) |
| Zinc, mg | 1154 | -0.05 (-0.08, -0.02) | (-1.0, 2.4) | 0.044 (0.005, 0.083) |
| Copper, mg | 1153 | -0.0001 (-0.003, 0.003) | (-0.1, 2.0) | 0.033 (-0.002, 0.067) |
| Manganese, mg | 1033 | -0.001 (-0.01, 0.01) | (-0.3, 2.2) | -0.009 (-0.05, 0.032) |
| Iodine, µg | 777 | 98.7 (29.2, 168.2) | (-1838.7, 1069.4) | 1.246 (1.151, 1.340) |
| Selenium, µg | 805 | -0.9 (-1.5, -0.4) | (-16.1, 8.6) | -0.321 (-0.362, -0.279) |
| Chromium, µg | 699 | -0.070 (-0.142, 0.003) | (-2.0, 2.9) | -0.119 (-0.188, -0.051) |
| Molybdenum, µg | 822 | 1.8 (0.9, 2.8) | (-24.8, 17.2) | 0.140 (0.103, 0.176) |
| Retinol, µg | 433 | -6.1 (-8.4, -3.8) | (-54.1, 19.9) | -0.247 (-0.322, -0.172) |
| α-carotene, µg | 412 | 19.6 (0.3, 39.0) | (-373.0, 217.9) | -0.151 (-0.233, -0.07) |
| β-carotene, µg | 784 | -47.7 (-86.0, -9.4) | (-1121.1, 491.0) | -0.172 (-0.222, -0.121) |
| Cryptoxanthin, µg | 381 | -0.9 (-2.1, 0.4) | (-25.1, 13.3) | -0.028 (-0.072, 0.015) |
| Vitamin D, µg | 512 | -0.4 (-0.6, -0.2) | (-4.9, 3.8) | -0.552 (-0.596, -0.507) |
| α-tocopherol, mg | 1043 | -0.08 (-0.12, -0.04) | (-1.3, 2.6) | -0.363 (-0.408, -0.319) |
| β-tocopherol, mg | 544 | -0.003 (-0.008, 0.002) | (-0.1, 2.1) | -0.272 (-0.323, -0.22) |
| γ-tocopherol, mg | 806 | -0.1 (-0.25, 0.04) | (-4.2, 3.9) | -0.501 (-0.564, -0.438) |
| δ-tocopherol, mg | 565 | -0.01 (-0.06, 0.04) | (-1.1, 2.5) | -0.331 (-0.408, -0.254) |
| Vitamin K, µg | 900 | -1.8 (-3.3, -0.3) | (-47.4, 23) | -0.064 (-0.096, -0.032) |
| Vitamin B_1_, mg | 1142 | -0.01 (-0.02, -0.01) | (-0.3, 2.1) | -0.410 (-0.472, -0.349) |
| Vitamin B_2_, mg | 1162 | -0.02 (-0.03, -0.01) | (-0.3, 2.1) | -0.643 (-0.694, -0.592) |
| Niacin, mg | 1162 | -0.20 (-0.26, -0.08) | (-3.2, 3.3) | -0.311 (-0.353, -0.27) |
| Vitamin B_6_, mg | 1153 | -0.01 (-0.02, -0.01) | (-0.2, 2.1) | -0.205 (-0.242, -0.167) |
| Vitamin B_12_, µg | 804 | -0.20 (-0.28, -0.05) | (-3.4, 3.5) | -0.556 (-0.601, -0.51) |
| Folate, µg | 1135 | -2.4 (-3.5, -1.3) | (-38.6, 17.7) | -0.102 (-0.138, -0.066) |
| Pantothenic acid, mg | 1140 | -0.03 (-0.05, -0.01) | (-0.7, 2.3) | -0.054 (-0.092, -0.016) |
| Biotin, µg | 831 | -0.5 (-0.8, -0.3) | (-7.0, 4.7) | -0.412 (-0.462, -0.362) |
| Vitamin C, mg | 800 | -1.5 (-2.2, -0.8) | (-20.4, 9.9) | -0.119 (-0.169, -0.069) |
| SFA, Saturated fatty acid, MUFA, Monounsaturated fatty acid, PUFA, Polyunsaturated fatty acid | | | | |
| ^*^ (Food photography method - weighed value) / (weighed value) × 100 | | | | |
| ^‡^ (Mean difference - 2SD, mean difference + 2SD) | | | | |
| ^⁋^ A linear regression model included difference as a dependent variable and mean as an independent variable. | | | | |
