# Supplementary material for: Development and validation of nutrient estimates based on a food-photographic record in Japan
Source: Nutr J. 2020 Sep 18;19:104. doi: 10.1186/s12937-020-00615-y (PMC7501716; doi:10.1186/s12937-020-00615-y)
Supplement: Supplementary file 3 — Additional file 3: List of the 44 nutrients measured the study. [file 12937_2020_615_MOESM3_ESM.docx]

**List of the 44 nutrients measured the study**

Energy, protein, fat, triglyceride, saturated fatty acid, monounsaturated fatty acid, polyunsaturated fatty acid, cholesterol, carbohydrate, total dietary fiber, water-soluble dietary fiber, water-insoluble dietary fiber, sodium, potassium, calcium, magnesium, phosphorus, iron, zinc, copper, manganese, iodine, selenium, chromium, molybdenum, retinol, α-carotene, β-carotene, cryptoxanthin, vitamin D, α-tocopherol, β-tocopherol, γ-tocopherol, δ-tocopherol, vitamin K, vitamin B1, vitamin B2, niacin, vitamin B6, vitamin B12, folate, pantothenic acid, biotin, and vitamin C.
